# Supplementary material for: Divergent Copies of a Cryptosporidium parvum-Specific Subtelomeric Gene
Source: Microorganisms. 2019 Sep 18;7(9):366. doi: 10.3390/microorganisms7090366 (PMC6780254; doi:10.3390/microorganisms7090366)
Supplement: Supplementary file 1 [file microorganisms-07-00366-s001.zip › supplementary-final/supplementary-final.docx]

Supplementary：


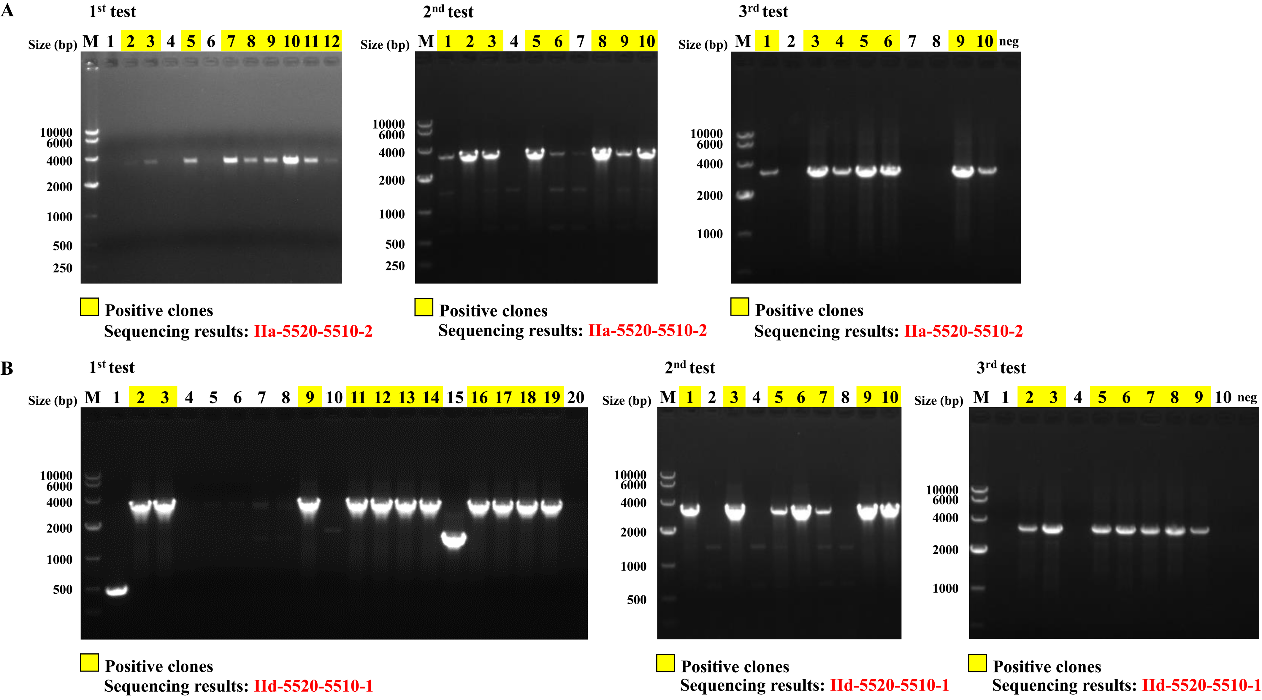


|  |
| --- |

**Figure 1.** Positive clones (those marked yellow) from three independent experiments of TA cloning of the *cgd6_5520-5510* gene in *Cryptosporidium parvum* IIa (**A**) and IId (**B**) subtypes. In each TA cloning experiment, the *cgd6_5520-5510* gene was amplified from IIa or IId genomic DNA and cloned into the pMD 18-T Vector. All the positive clones were identified by PCR and sequencing. We obtained a total of 20 positive clones of the IIa-5520-5510-2 copy and 25 positive clones of the IId-5520-5510-1 copy from three individual experiments.

| 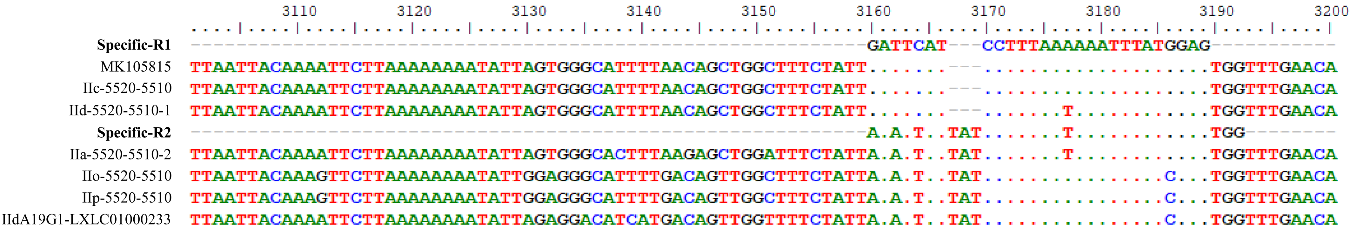 |
| --- |

**Figure 2.** The location and sequence differences of the copy-specific reverse primers in the alignment of *cgd6_5520-5510* sequences from *Cryptosporidium parvum* IIa, IId, IIc, IIo, and IIp subtypes. Specific-R1 is identical to MK105815 (IIa-5520-5510-1) and IIc-5520-5510 sequences, and specific-R2 is identical to IIa-5520-5510-2 sequence in the primer region. The IIdA19G1-LXLC01000233 sequence is identical to IId-5520-5510-2 sequence. The nucleotides identical to those in the reference sequence (the first line of the alignment) are shown as dots.


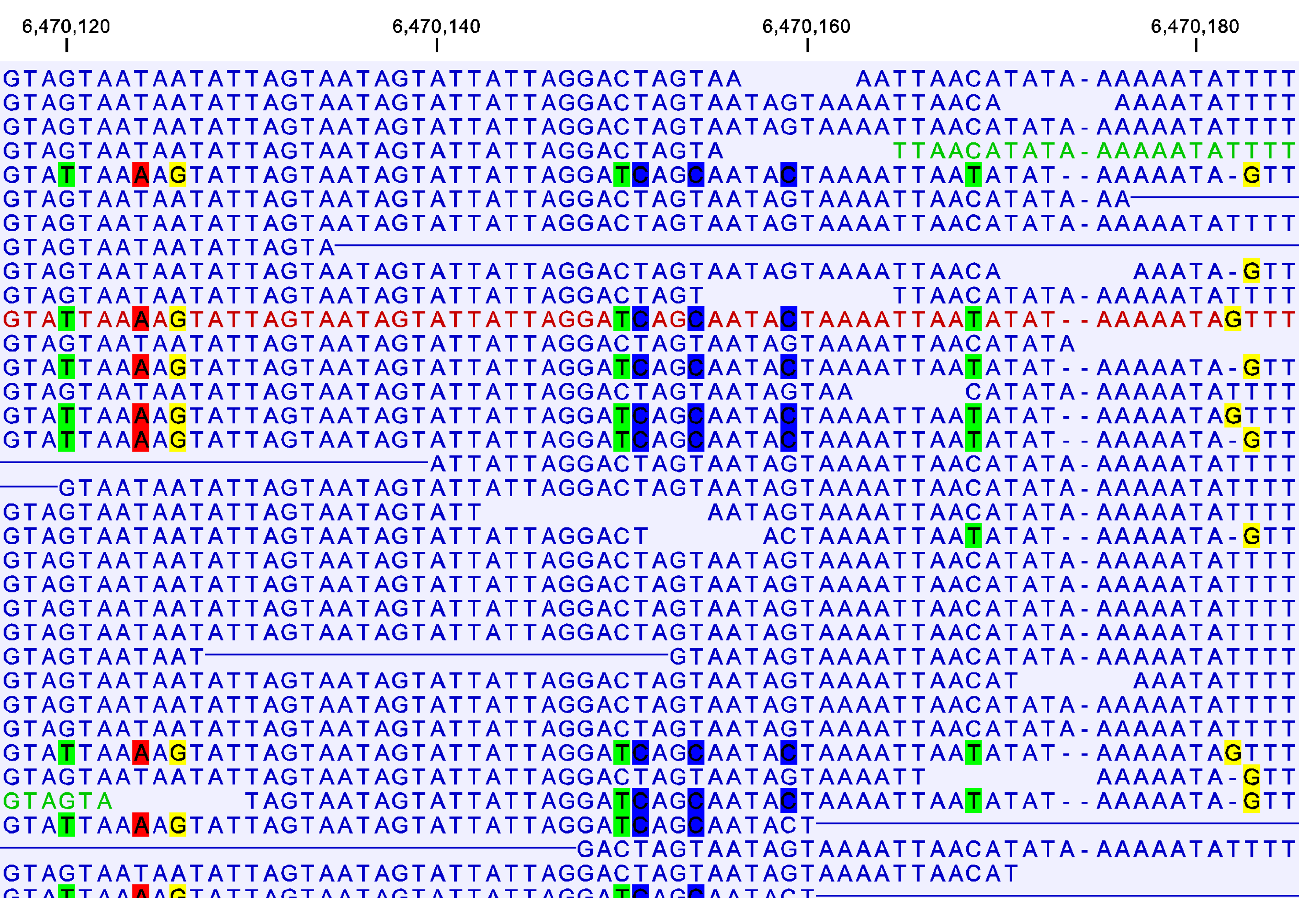


**Figure 3.** The presence of two copies of the *cgd6_5520-5510* gene in the IOWA isolate as revealed by mapping of Illumina sequence reads from whole genome sequencing of the isolate to the reference IOWA genome. Paired-end sequence reads (linked by a horizontal line between them) were mapped to eight chromosomes of the reference IOWA genome (AAEE00000000.1) linked together. The numbers above the sequences are nucleotide positions in the reference genome, which correspond to the *cgd6_5510* part of the *cgd6_5520-5510* gene in chromosome 6. Nucleotides different from the reference genome are highlighted in color.

| 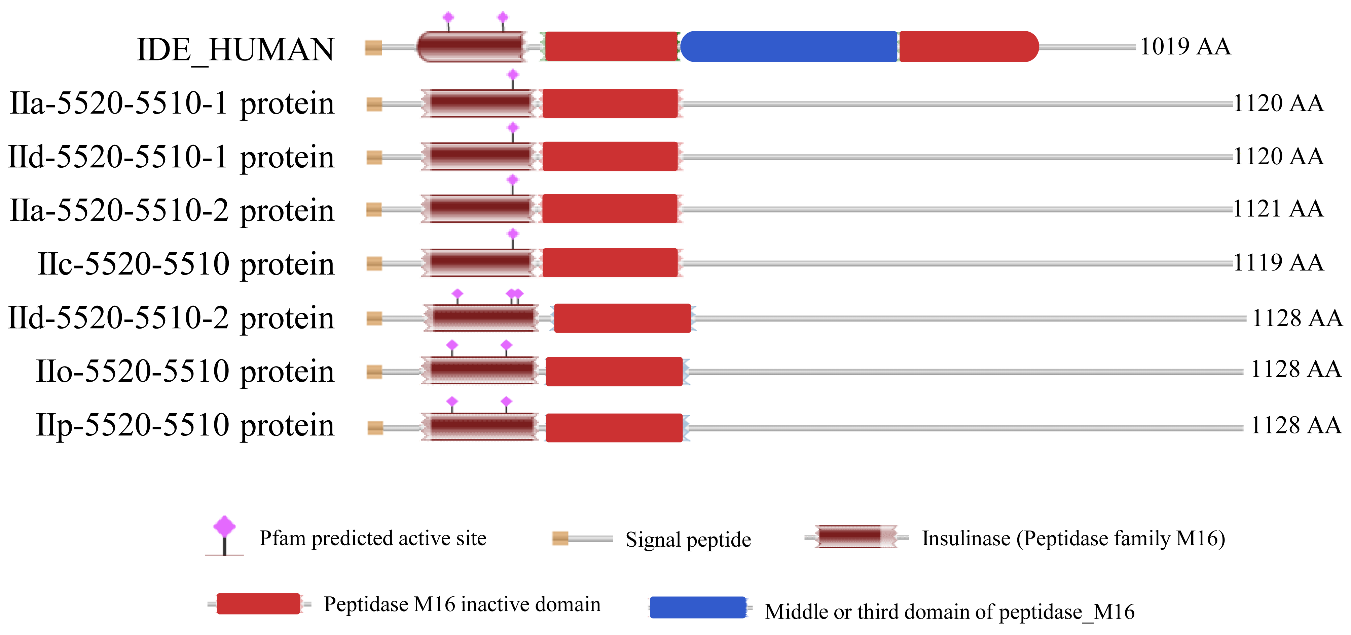 |
| --- |

**Figure** **S4.** A schematic representation of the domain structure of the *cgd6_5520-5510* gene in *Cryptosporidium parvum* IIa, IId, IIc, IIo, and IIp subtype families. The domain structure analysis revealed that all the proteins encoded by the *cgd6_5520-5510* gene had only two of the four domains in classic insulinases (such as IDE_HUMAN). IDE_HUMAN is an insulinase sequence from humans. The domains of the protein were predicated by using HMMER (https://www.ebi.ac.uk/Tools/hmmer/search/hmmscan) and drawn to scale.
