# Supplementary material for: Divergent Copies of a Cryptosporidium parvum-Specific Subtelomeric Gene
Source: Microorganisms. 2019 Sep 18;7(9):366. doi: 10.3390/microorganisms7090366 (PMC6780254; doi:10.3390/microorganisms7090366)
Supplement: Supplementary file 1 [file microorganisms-07-00366-s001.zip › supplementary-final/microorganisms-578516-tables.doc]

**Table S1.** Sequencing results for positive clones of the *cgd6_5520-5510* gene in *Cryptosporidium parvum* IIa subtype from three independent experiments.

| **Test** | **No.** | **PCR Product Tube#** | **Seq result** | **Sequencing region** | **Description** |
| --- | --- | --- | --- | --- | --- |
| 1 | 1 | 2 | IIa-5520-5510-2 | Full length | Identical |
|  | 2 | 3 | IIa-5520-5510-2 | Full length | Identical |
|  | 3 | 6 | IIa-5520-5510-2 | Full length | 2 base insertions |
|  | 4 | 13 | IIa-5520-5510-2 | Full length | 4 base substitutions, 1 base insertion |
|  | 5 | 14 | IIa-5520-5510-2 | Full length | 1 base deletion |
|  | 6 | 15 | IIa-5520-5510-2 | Full length | 2 base substitutions, a 3-base deletion |
|  | 7 | 16 | IIa-5520-5510-2 | Full length | Identical |
|  | 8 | 17 | IIa-5520-5510-2 | Full length | Identical |
|  | 9 | 18 | IIa-5520-5510-2 | Full length | Identical |
| 2 | 10 | 1 | IIa-5520-5510-2 | Full length | 1 base deletion |
|  | 11 | 2 | IIa-5520-5510-2 | Full length | Identical |
|  | 12 | 3 | IIa-5520-5510-2 | Full length | 1 base substitution, 1 base deletion |
|  | 13 | 5 | IIa-5520-5510-2 | Full length | Identical |
|  | 14 | 6 | IIa-5520-5510-2 | Full length | Identical |
|  | 15 | 8 | IIa-5520-5510-2 | Full length | 2 base substitutions |
|  | 16 | 9 | IIa-5520-5510-2 | Full length | Identical |
|  | 17 | 10 | IIa-5520-5510-2 | Full length | Identical |
| 3^*^ | 18 | 1 | IIa-5520-5510-2 | ~1000 bp at 3´ end | Identical |
|  | 19 | 3 | IIa-5520-5510-2 | ~1000 bp at 3´ end | Identical |
|  | 20 | 4 | IIa-5520-5510-2 | ~1000 bp at 3´ end | Identical |
|  | 21 | 5 | IIa-5520-5510-2 | ~1000 bp at 3´ end | Identical |
|  | 22 | 6 | IIa-5520-5510-2 | ~1000 bp at 3´ end | Identical |
|  | 19 | 9 | IIa-5520-5510-2 | ~1000 bp at 3´ end | Identical |
|  | 20 | 10 | IIa-5520-5510-2 | ~1000 bp at 3´ end | Identical |

^*^ After we obtained the full length of one of the two *cgd6_5520-5510* copies in the IIa or IId subtype, we then sequenced the 3´ end of the gene instead of the full length to determine the sequence results.

**Table S2.** Sequencing results for positive clones of the *cgd6_5520-5510* gene in *Cryptosporidium parvum* IId subtype from three independent experiments.

| **Test** | **No.** | **PCR Product Tube#** | **Seq result** | **Sequencing region** | **Description** |
| --- | --- | --- | --- | --- | --- |
| 1 | 1 | 2 | IId-5520-5510-1 | Full length | Identical |
|  | 2 | 3 | IId-5520-5510-1 | Full length | 3 base substitutions and 1 base deletion |
|  | 3 | 9 | IId-5520-5510-1 | Full length | Identical |
|  | 4 | 11 | IId-5520-5510-1 | Full length | 2 base substitutions and 1 base deletion |
|  | 5 | 12 | IId-5520-5510-1 | Full length | Doublet at 5´ end |
|  | 6 | 13 | IId-5520-5510-1 | Full length | Identical |
|  | 7 | 14 | IId-5520-5510-1 | Full length | 1 base substitution |
|  | 8 | 16 | IId-5520-5510-1 | Full length | 1 base substitution and 2 base deletions |
|  | 9 | 17 | IId-5520-5510-1 | Full length | Identical |
|  | 10 | 18 | IId-5520-5510-1 | Full length | Identical |
|  | 11 | 19 | IId-5520-5510-1 | Full length | a 3-base deletion |
| 2 | 12 | 1 | IId-5520-5510-1 | Full length | Identical |
|  | 13 | 2 | IId-5520-5510-1 | Full length | Identical |
|  | 14 | 4 | IId-5520-5510-1 | Full length | 1 base substitution |
|  | 15 | 5 | IId-5520-5510-1 | Full length | Identical |
|  | 16 | 6 | IId-5520-5510-1 | Full length | Identical |
|  | 17 | 8 | IId-5520-5510-1 | Full length | Identical |
|  | 18 | 10 | IId-5520-5510-1 | Full length | Identical |
| 3^*^ | 19 | 2 | IId-5520-5510-1 | ~1000 bp at 3´ end | Identical |
|  | 20 | 3 | IId-5520-5510-1 | ~1000 bp at 3´ end | Identical |
|  | 21 | 5 | IId-5520-5510-1 | ~1000 bp at 3´ end | Identical |
|  | 22 | 6 | IId-5520-5510-1 | ~1000 bp at 3´ end | Identical |
|  | 23 | 7 | IId-5520-5510-1 | ~1000 bp at 3´ end | Identical |
|  | 24 | 8 | IId-5520-5510-1 | ~1000 bp at 3´ end | Identical |
|  | 25 | 9 | IId-5520-5510-1 | ~1000 bp at 3´ end | Identical |

^*^ After we obtained the full length of one of the two *cgd6_5520-5510* copies in the IIa or IId subtype, we then sequenced the 3´ end of the gene instead of the full length to determine the sequence results.
